# Flavonoids from mulberry leaves: physiological functions, molecular mechanisms and applications in juvenile animal health—A review

**DOI:** 10.3389/fvets.2026.1903941

**Published:** 2026-07-22

**Authors:** Ying-Ying Liu, Wen-Xin Jiang, Yu Chen, Hua-Li Li, Yu-Qing Sun, Hui-Bo Ren, Xiong-Gui Hu, Chen Chen, Wei-Min Jiang, Yi Xiao, Rui-Hua Huang, Jia-Shun Chen, Ying-Lin Peng, You-Gui Li, Yu-Long Yin

**Affiliations:** 1 Hunan Institute of Animal and Veterinary Science, Changsha, China; 2 Yuelushan Laboratory, Changsha, Hunan, China; 3 College of Animal Science and Technology, Hunan Agricultural University, Changsha, Hunan, China; 4 State Key Laboratory for Quality and Safety of Agro-Products, Institute of Sericulture and Tea, Zhejiang Academy of Agricultural Sciences, Hangzhou, China; 5 College of Information and Intelligence, Hunan Agricultural University, Changsha, China; 6 Institute of Swine Science (Key Laboratory of Pig Genetic Resources Evaluation and Utilization, Ministry of Agriculture and Rural Affairs (Nanjing)), Nanjing Agricultural University, Nanjing, China

**Keywords:** anti-inflammatory, antioxidant, flavonoids from mulberry leaves, intestinal health, juvenile animal

## Abstract

The intensive use of antibiotics in juvenile livestock and aquaculture has led to serious public health risks such as drug residues, bacterial resistance, and environmental pollution, creating an urgent need for safe and efficient antibiotic alternatives. Flavonoids from mulberry leaves (FML), as typical natural polyphenolic compounds, exhibit strong antioxidant, anti-inflammatory, immunomodulatory, intestinal microbiota-regulating, and metabolic-improving activities, showing great potential in promoting juvenile animal health. This review sorts out global published literature concerning FML biological functions and animal feeding trials to clarify its multi-target regulatory mechanisms and practical application value in young animals. We integrated research data covering weaned piglets, calves, juvenile fish and broiler chicks, summarized core signaling pathways mediated by FML, and consolidated *in vivo* phenotypic effects observed across different juvenile animal models. Mechanistic analysis demonstrates that FML activates the Nrf2 pathway to alleviate oxidative damage, inhibits NF-κB/TLR4/MAPK inflammatory cascades, stabilizes intestinal barrier integrity and microbiota homeostasis, and regulates IRS-1/PI3K/GLUT4 and PPARα pathways to optimize glucose and lipid metabolism. Feeding trials further confirm that FML supplementation effectively mitigates weaning stress, lowers diarrhea rates, enhances non-specific immunity and improves overall growth performance of juvenile animals. Nevertheless, widespread application of FML is still constrained by unresolved issues including inconsistent optimal dosages across species, low *in vivo* bioavailability, and insufficient large-scale farm verification data. This paper summarizes these critical limitations and proposes feasible directions for subsequent mechanistic and applied research. Overall, this review offers comprehensive theoretical references for the development and popularization of FML as green feed additives within sustainable livestock and aquaculture systems.

## 1 Introduction

With the rapid expansion of global animal husbandry and aquaculture, the demand for meat products has increased dramatically. To improve growth efficiency, antibiotics have been widely used as growth promoters in juvenile animal production, especially in pigs, cattle, poultry, and fish at the neonatal, suckling, or weaning stages (1). Due to the immature development of digestive, immune, thermoregulatory and metabolic functions in young animals, they exhibit fragile intestinal barrier integrity, low immune competence, high energy metabolic rate and poor thermoregulatory capacity. Meanwhile, young animals are highly susceptible to environmental and nutritional stress, and their early growth and health status are heavily dependent on colostrum intake and appropriate nutritional supply (2, 3). To improve the survival rate of young animals and enhance economic benefits, intensive farms extensively use antibiotics to counteract the physiological deficiencies of young animals, including underdeveloped digestive function, low immune competence, and vulnerable intestinal tract susceptible to pathogenic bacterial invasion. However, long-term overuse of antibiotics has triggered a series of challenges, including antimicrobial resistance, drug residues in animal products, and ecological contamination, which pose major threats to public health and food security. In recent years, numerous countries and regions have introduced policies to restrict or ban the use of antibiotics as feed additives, prompting the industry to seek safe, sustainable, and efficient natural alternatives.

Against this background, green and natural plant-derived feed additives have become a research hotspot in sustainable animal husbandry. Researchers have attempted to develop various alternative additives to replace traditional antibiotics for improving animal growth performance and stress resistance. Nevertheless, each type of substitute possesses unique advantages as well as unavoidable application defects, limiting its large-scale and long-term promotion in actual production. With the comprehensive ban on antibiotic growth promoters in livestock industry, a variety of green feed additives have been widely developed and applied to alleviate oxidative stress, inhibit inflammatory response and improve animal production performance, among which probiotics, organic acids, essential oils, tannins and diverse plant polyphenols are the most representative categories. Each type of feed additive has its own application characteristics and inherent limitations.

Probiotics exert regulatory effects mainly by optimizing intestinal flora structure, but their activity is easily affected by feed pelleting high temperature, gastric acid and bile salt erosion, resulting in unstable application effects (4). Organic acids can lower intestinal pH value to inhibit the proliferation of harmful bacteria, yet they only show weak antioxidant and anti-inflammatory activities, and excessive addition will damage the intestinal mucosal structure of young animals (5). Plant essential oils have strong bacteriostatic and anti-inflammatory properties, while they are volatile, prone to oxidation and deterioration during feed storage, accompanied by peculiar odor that may reduce animal feed intake (6). Tannins possess excellent antioxidant capacity, but high-dose supplementation easily leads to the inhibition of digestive enzyme activity, reducing the apparent digestibility of protein and carbohydrates (7).

Given the limitations of the above common antibiotic substitutes, plant extracts have gradually attracted extensive research attention owing to their natural, non-toxic, residue-free advantages and multi-target biological regulatory functions, which can simultaneously alleviate oxidative damage, suppress excessive inflammatory responses and optimize intestinal health in young animals. Among numerous plant-derived active substances, mulberry leaf extracts stand out due to abundant raw material sources, low production cost and excellent biological safety, making them a promising candidate for novel green feed additives.

In China, mulberry has a cultivation history of over 4,700 years, initially for sericulture to nourish silkworms, and has since been widely utilized in traditional medicine, food, and animal feed. The leaves are rich in proteins, carbohydrates, vitamins, minerals, and dietary fiber, and also contain bioactive compounds, such as flavonoids, alkaloids, and polysaccharides, which contribute to their health-promoting properties (8). Among these bioactive constituents, FML—primarily rutin, isoquercitrin, astragalin, and nicotiflorin—have been confirmed to possess diverse biological activities, including antioxidant, anti-inflammatory (9), gut microbiota-regulation (10), hypolipidemic effect (11), hypoglycemic effect (12), anti-tumor activity (13), and reduction of obesity risk (14, 15). Notably, mulberry leaves have been used in traditional Chinese medicine for over 3,000 years to treat conditions such as colds and diabetes, and have been recognized as dual-purpose pharmaceutical and food resources by the Ministry of Public Health of China (8). Their high protein content and digestibility also make them a valuable alternative feed resource for sustainable livestock production (16).

Among the various bioactive constituents of mulberry leaves, FML are particularly notable for their diverse biological activities and potential health benefits. FML, a group of polyphenolic compounds such as rutin, isoquercitrin, nicotiflorin, and astragalin (17), are widely distributed in the plant kingdom. In detail, Ju et al. (18) identified a total of 17 FML in mulberry leaves and quantified their concentrations using high-performance liquid chromatography. Similarly, Kim et al. (19) isolated 9 flavonoid compounds from mulberry leaves and identified isoquercitrin, rutin, and astragalin as the predominant components with strong antioxidant activity. Flavonoids are known for their ability to modulate key cellular enzymes and signaling pathways, which underscores their therapeutic potential in preventing and managing various chronic diseases, including cardiovascular disorders, cancers, and neurodegenerative conditions (20).

This review provides a comprehensive overview of the physiological functions of FML derived from mulberry and explores their potential applications in juvenile animal health. The aim is to provide a reference for the development and application of FML as functional feed additives in modern animal husbandry.

## 2 Extraction methods for flavonoids from mulberry leaves

Currently, the most commonly used methods for extracting FML from *Morus alba* include conventional solvent extraction, ultrasound-assisted extraction, supercritical CO₂ extraction, and microwave-assisted extraction. For purification, macroporous resin adsorption is frequently employed. Table 1 summarizes the common extraction and purification methods for FML, along with their respective advantages and disadvantages.

**Table 1:** Extraction and purification methods of flavonoids from mulberry leaves.

Methodology	Advantage	Disadvantage	Reference
Traditional solvent extraction	Straightforward procedures; commercially available low-cost solvents; mature technology suitable for large-scale industrial promotion.	Large solvent dosage; lengthy extraction time; relatively low flavonoid extraction yield; conventional organic solvents present flammability risks requiring strict explosion-proof workshop configuration.	(48, 49)
Ultrasound-assisted extraction	Ultrasound cavitation disrupts plant cell walls, greatly shortening extraction time; reduced solvent consumption; elevated recovery efficiency of target FML.	Long-duration high-power ultrasonic treatment may trigger oxidative degradation of partial heat-sensitive polyphenolic components; prolonged direct exposure to high-intensity ultrasound induces mild physical discomfort to operators without proper sound insulation.	(50)
Supercritical CO_2_ extraction	Short processing cycle; CO₂ is non-toxic, odorless and recyclable; mild low-temperature environment minimizes structural damage to bioactive FML.	High upfront investment in high-pressure equipment; poor affinity for strongly polar flavonoid monomers and high-molecular-weight polyphenols, requiring modifier addition to improve recovery.	(51)
Microwave-assisted extraction	Rapid, selective extraction; short heating duration preserves the native biological activity of FML.	Inefficient recovery of bound phenolic FML; aging microwave equipment with inadequate shielding may produce trace radiation leakage under long-term continuous operation.	(52, 53)
Macroporous resin adsorption	Excellent adsorption capacity and separation efficiency for flavonoid monomers; effective decolorization and impurity removal to improve product purity.	Strict pretreatment requirements for crude extracts; resin performance declines drastically under extreme high temperature, strong acid or alkaline conditions; unfavorable for small-batch laboratory extraction.	(54, 55)

## 3 Chemical constituents and structures of flavonoids

Flavonoids constitute a large group of plant secondary metabolites characterized by a typical C₆-C₃-C₆ carbon skeleton, which consists of two benzene rings (Ring A and Ring B) linked by a three-carbon chain (Ring C), and are derived from the phenylpropanoid metabolic pathway. Ring A and Ring B are substituted with various functional groups, such as hydroxyl, methoxyl, and prenyl groups, which collectively contribute to their structural diversity and classification. Based on the oxidation state and degree of unsaturation of Ring C, as well as the substitution position of Ring B, flavonoids are classified into several subclasses, including flavones, flavonols, isoflavones, anthocyanidins, and chalcones. The detailed chemical composition, core structural characteristics, and typical subclasses of flavonoids derived from mulberry leaves (9) are summarized in Table 2.

**Table 2:** Chemical constituents and structures of flavonoids.

Flavonoid subclasses	Core chemical constituents (skeleton, substituents and natural forms)	Key structural characteristics	Main activity characteristics (*in vitro*/*in vivo* activities with typical research links)	Typical representative compounds	Main references
Flavones	Skeleton: C₆-C₃-C₆ (15 carbons); Ring A is mostly resorcinol/phloroglucinol type, Ring B is phenyl group; Common substituents include hydroxyl (-OH), methoxyl (-OCH₃), and some glycosyl groups (glucose, rhamnose, etc.); Natural forms are mainly O-glycosides, with a small amount of free aglycones.	Ring C is *γ*-pyrone structure, with C₂ = C₃ double bond and C₄ = O; no C₃-OH; Ring B is attached to C₂ position; Ring A usually contains hydroxyl groups at C₅ and C₇.	1. Antioxidant activity: Scavenges DPPH and ABTS free radicals, and inhibits lipid peroxidation; 2. Anti-inflammatory activity: Inhibits the release of inflammatory factors such as NO, TNF-*α* and IL-6; 3. Antibacterial activity: Inhibits Gram-positive bacteria and fungi; 4. Some have hepatoprotective and neuroprotective activities.	Apigenin, Luteolin, Chrysin, Apigenin-7-O-glucoside	(56, 57)
Flavonols	Skeleton: C₆-C₃-C₆ (15 carbons); Ring A is mostly phloroglucinol type, Ring B is phenyl group; Substituents are mainly hydroxyl, methoxyl and glycosyl groups, and some contain prenyl groups; Natural forms include free aglycones and O-glycosides (glycosylation at C₃ and C₇ is common).	Ring C is *γ*-pyrone structure, with C₂ = C₃ double bond and C₄ = O; the key feature is the presence of hydroxyl group at C₃ (C₃-OH); Ring B is attached to C₂ position; Ring A usually contains hydroxyl groups at C₅ and C₇, and Ring B mostly contains hydroxyl groups at C₃’ and C₄’.	1. Strong antioxidant activity: Its antioxidant capacity is superior to most flavonoid subclasses, and it can chelate metal ions; 2. Anti-inflammatory and anti-tumor activities: Inhibits tumor cell proliferation, induces apoptosis, and alleviates inflammatory responses; 3. Cardiovascular protection: Regulates blood lipids and improves vascular endothelial function; 4. Neuroprotection: Reduces oxidative damage to nerve cells.	Quercetin, Kaempferol, Myricetin, Rutin, Isorhamnetin	(58, 59)
Flavanones	Skeleton: C₆-C₃-C₆ (15 carbons); Ring A is mostly resorcinol type, Ring B is phenyl group; Substituents are mainly hydroxyl, methoxyl and glycosyl groups; Natural forms are mostly O-glycosides (common at C₇), with a small amount of free aglycones, and chiral isomers exist.	Ring C is γ-pyrone structure, with C₂-C₃ single bond (saturated) and C₄ = O; Ring B is attached to C₂ position; Ring A usually contains hydroxyl groups at C₅ and C₇; C₂ is a chiral carbon (with R/S configuration).	1. Antioxidant and anti-inflammatory activities: Scavenges free radicals and inhibits the expression of inflammatory factors; 2. Hepatoprotective activity: Reduces hepatocyte damage and promotes hepatocyte repair; 3. Lipid-regulating and hypoglycemic activities, and some have antidepressant effects.	Naringenin, Hesperetin, Naringin, Hesperidin	(60, 61)
Flavanonols	Skeleton: C₆-C₃-C₆ (15 carbons); Ring A is mostly phloroglucinol type, Ring B is phenyl group; Substituents are mainly hydroxyl and methoxyl groups, and some contain glycosyl groups; Natural forms are free aglycones or O-glycosides, and chiral isomers exist.	Ring C is γ-pyrone structure, with C₂-C₃ single bond (saturated) and C₄ = O; the key feature is the presence of hydroxyl group at C₃ (C₃-OH); both C₂ and C₃ are chiral carbons, and Ring B is attached to C₂ position.	1. Strong antioxidant activity: It has significant ability to scavenge free radicals and protect cells from oxidative damage; 2. Anti-inflammatory and anti-tumor activities: Inhibits tumor cell migration and alleviates inflammatory responses; 3. Neuroprotective activity: Improves nerve cell survival and delays neurodegenerative diseases.	Taxifolin, Dihydroquercetin, Dihydrokaempferol	(62, 63)
Isoflavonoids	Skeleton: C₆-C₃-C₆ (15 carbons); Ring A is resorcinol type, Ring B is phenyl group; Substituents are mainly hydroxyl, methoxyl and glycosyl groups; Natural forms are mostly O-glycosides, with a small amount of free aglycones, mainly existing in leguminous plants.	Ring C is γ-pyrone structure, with C₂ = C₃ double bond and C₄ = O; the key feature is that Ring B is attached to C₃ position; Ring A usually contains hydroxyl groups at C₅ and C₇, and some have chiral isomers.	1. Phytoestrogen activity: Regulates estrogen levels in the body and improves osteoporosis; 2. Antioxidant and anti-inflammatory activities: Scavenges free radicals and inhibits inflammatory responses; 3. Anti-tumor activity: Inhibits the proliferation of breast cancer and prostate cancer cells; 4. Cardiovascular protective activity.	Genistein, Daidzein, Genistin, Daidzin	(64, 65)
Flavan-3-ols	Skeleton: C₆-C₃-C₆ (15 carbons); Ring A is resorcinol/phloroglucinol type, Ring B is phenyl group; Substituents are mainly hydroxyl groups, and some contain glycosyl groups; Natural forms are free aglycones, with a large number of oligomers (such as proanthocyanidins), and significant chirality.	Ring C is pyran ring structure, with C₂-C₃ single bond (saturated) and no C₄ = O; the key feature is the presence of hydroxyl group at C₃ (C₃-OH); both C₂ and C₃ are chiral carbons (with multiple configurations), and Ring B is attached to C₂ position.	1. Extremely strong antioxidant activity: It has the highest antioxidant capacity among flavonoids, which can scavenge free radicals and chelate metal ions; 2. Anti-inflammatory and anti-tumor activities: Inhibits tumor angiogenesis and alleviates inflammatory responses; 3. Cardiovascular protection: Lowers blood lipids and inhibits platelet aggregation.	(+)-Catechin, (−)-Epicatechin, Epigallocatechin Gallate (EGCG), Proanthocyanidins	(66, 67)
Chalcones	Skeleton: C₆-C₃-C₆ (15 carbons); two benzene rings (Ring A and Ring B) are connected by α,β-unsaturated ketone; Substituents are mainly hydroxyl, methoxyl and prenyl groups; Natural forms are mostly free aglycones, and some can be converted into flavanones.	Ring C is an open-loop structure, containing α,β-unsaturated ketone (-CO-CH=CH-); Ring A and Ring B are directly attached to both ends of the unsaturated carbon chain, without a closed pyrone structure.	1. Antioxidant and anti-inflammatory activities: Scavenges free radicals and inhibits the release of inflammatory factors; 2. Anti-tumor and antibacterial activities: Inhibits tumor cell growth and has inhibitory effects on a variety of pathogenic bacteria; 3. Hepatoprotective and antiviral activities.	Isoliquiritigenin, Liquiritigenin, Chalcone A	(68, 69)

## 4 Physiological functions of FML

### 4.1 Antioxidant function

Oxidative stress occurs when an organism is exposed to harmful stimuli, leading to the excessive production of highly reactive molecules such as reactive oxygen species (ROS) and reactive nitrogen species (RNS) (21). When the rate of oxidation exceeds the capacity of antioxidant defense mechanisms, it results in an imbalance between oxidative and antioxidative processes, causing tissue damage (22). As natural antioxidants, flavonoids enhance the activities of superoxide dismutase (SOD) and catalase (CAT), reduce malondialdehyde (MDA) levels, scavenge ROS, chelate metal ions, and inhibit lipid peroxidation, thus strengthening antioxidant capacity and protecting cells from oxidative damage (23, 24). Liu et al. (25) reported that dietary supplementation with the compound of FML and carnosic acid upregulated the mRNA expression of PGC-1α, Nrf2 and Keap1 in the jejunum of broiler chicks. Moreover, the 150 mg/kg combined additive group markedly increased the transcriptional levels of p38, PGC-1α, Nrf2 and Keap1 in the ileum, thereby activating the p38 MAPK/Nrf2 antioxidant signaling pathway. FML exerts strong antioxidant effects mainly by activating the Nrf2-ARE pathway. Under oxidative stress, FML promotes the dissociation of nuclear factor erythroid 2–related factor 2 (Nrf2) from Keap1 and its nuclear translocation, where Nrf2 binds to ARE and upregulates transcription of antioxidant genes including SOD, CAT, glutathione peroxidase (GSH-Px) and heme oxygenase-1 (HO-1). Meanwhile, FML scavenges ROS, reduces MDA accumulation and stabilizes mitochondrial membrane potential, thereby alleviating oxidative damage in young and weaned animals caused by weaning, heat stress or toxin challenge (10, 26, 27).

### 4.2 Anti-inflammatory function

Inflammation is a fundamental pathological process that occurs when tissues are exposed to harmful stimuli, such as trauma, bacterial infection, or other damaging factors, serving primarily as a defense mechanism manifested by redness, swelling, heat, and pain. Several studies have reported the potential of flavonoids as anti-inflammatory agents (28). In the central nervous system, FML enhance neuronal survival and synaptic plasticity by promoting brain-derived neurotrophic factor (BDNF) and reducing neuroinflammation (29). Besides, FML regulate mitogen-activated protein kinases (MAPKs), activator protein-1 (AP-1), peroxisome proliferator-activated receptor alpha (PPAR*α*), nuclear factor kappa-light-chain-enhancer of activated B cells (NF-κB), and Nrf2 to exert anti-inflammatory and antioxidant effects (30). Flavonoids also exhibits notable anti-inflammatory properties by suppressing the production of pro-inflammatory cytokines, such as tumor necrosis factor-alpha (TNF-α) and interleukin-6 (IL-6), and by inhibiting the cyclooxygenase (COX) and lipoxygenase activities via modulating intracellular signaling pathways, including NF-κB and MAPKs (31). IL-6 is a marker of chronic inflammation, which disrupts insulin signaling by inhibiting tyrosine kinase activity and activating suppressor of cytokine signaling 3 (SOCS3) (32). Duan et al. (33) demonstrated that FML inhibit the activity of key inflammatory signaling proteins, including toll-like receptor 4 (TLR4), myeloid differentiation primary response 88 (MyD88), tumor necrosis factor receptor-associated factor 6 (TRAF6), inhibitor of NF-κB alpha (I-κB*α*), phosphorylated I-κBα (p-I-κBα), NF-κB p65, and reduce IL-1β, IL-6, and TNF-α to alleviate liver inflammation associated with type 2 diabetes mellitus (T2DM). Furthermore, Shi et al. (34) found dietary FML (300 mg/kg) in high-carb eels enhance the activities of CAT and GSH-Px. This treatment also upregulates the mRNA expression of anti-inflammatory cytokines IL-10 and transforming growth factor-beta 1 (TGF-β1) by inhibiting the NF-κB/TLR signaling pathway. Concurrently it reduces serum levels of aspartate aminotransferase (AST), alanine aminotransferase (ALT), and MDA, as well as the mRNA expression of pro-inflammatory markers IL-1β, IL-8, and IL-12β to mitigate liver inflammation.

### 4.3 Regulation of the intestinal microbiota

Damage to the intestinal barrier leads to dysregulation of the intestinal microbial ecosystem and can ultimately result in extraintestinal diseases (35). Flavonoids can maintain the diversity of rumen microorganisms, improve microbial fermentation activity and inhibit methane production, while also regulating intestinal epithelial cell renewal, repairing intestinal mucosal barrier damage, and balancing the intestinal flora structure (36, 37). Studies have reported that dietary supplementation with 45 g/day of FML in buffaloes significantly increases the abundance of the intestinal phyla Bacteroidetes, Firmicutes, and Proteobacteria; promotes the proliferation of Encephalitozoon, Klebsiella, and Vibrio pseudobutyricus in the rumen; and maintains microbial homeostasis, allowing the animals to better adapt to hot and humid environments (38). Bi et al. (39) demonstrated that supplementation with 3 g/day of FML in calves enhances the relative abundance of beneficial intestinal bacteria, such as Lactobacillus and Enterococcus, increases total bacterial counts, and boosts the copy numbers of Prevotella and Lactobacillus in the jejunal chyme. These changes improved microbial balance, reduced diarrhea incidence, and enhanced intestinal health. Similarly, Zhu et al. (40) conducted an experiment by adding 2.5–10.0 mg/L quercetin to the water body for 21 days to treat *Odontobutis potamophila*. It was found that the low concentration of quercetin significantly increases the abundance of beneficial bacteria such as Bacillus and Lactobacillus in the intestinal tract, while reducing the number of pathogenic bacteria such as Aeromonas and Shewanella. This, in turn, maintains the balance of the intestinal flora of *Odontobutis potamophila* and improves the intestinal barrier function. Zhong et al. (41) observed that supplementation with 0.24 mg/day of FML in mice with a high-fat diet significantly improves the composition of the gut microbiota, reduces the Firmicutes to Bacteroidetes ratio, and increases the relative abundance of Clostridium, thereby enhancing acetic acid production and alleviating lipid metabolism disorders associated caused by high-fat diets. Additionally, Ma et al. (42) reported that the inclusion of 2 g/day of FML in sheep rations improves apparent nitrogen digestibility, maintains the balance of gut microbiota, and reduces nitrogen and methane emissions by inhibiting methanogenic microorganisms.

### 4.4 Reduction in blood glucose and blood lipid levels

Flavonoids have been shown to improve insulin resistance and lower blood cholesterol and glucose levels, thereby ensuring overall animal health (43). For instance, flavonoids supplementation improves insulin sensitivity in diabetic rats with decreased blood glucose, triglycerides (TG), total cholesterol (TC), and low-density lipoprotein (LDL) levels. These beneficial effects are mediated through the activation of the IRS-1, PI3K, and GLUT-4 signaling pathways, which enhance glucose uptake and metabolism in skeletal muscle (44). FML also enhance insulin sensitivity, promote glucose uptake by upregulating GLUT4 expression, and reduce lipid accumulation in adipocytes (45). Similarly, Zhong et al. (41) reported that administering 240 mg/kg/day of FML to mice with a high-fat diet (HFD) significantly decreases the average adipocyte size in epididymal white adipose tissue and reduces serum TG levels. Moreover, flavonoid treatment downregulates the mRNA expression levels of key lipid metabolism genes in mice, including ACC, PPARα, SREBP1, and SREBP2, contributing to reduced body weight gain and adiposity in HFD-fed mice. Huang et al. (46) further found that FML improve hepatic lipid metabolism and enhance laying hen performance by decreasing serum levels of total cholesterol (T-CHO), low-density lipoprotein cholesterol (LDL-C), and alkaline phosphatase (AKP), and increasing HDL-C levels and promoting hepatic fatty acid *β*-oxidation via upregulation of PPARα and CPT-I mRNA expression. In another study, Feng et al. (47) observed that dietary FML increase levels of polyunsaturated fatty acids and decrease saturated fatty acids in both liver and kidney tissues, resulting in a more favorable fatty acid profile lipid metabolism in Bama miniature pigs during the fattening stage.

**Figure 1:**
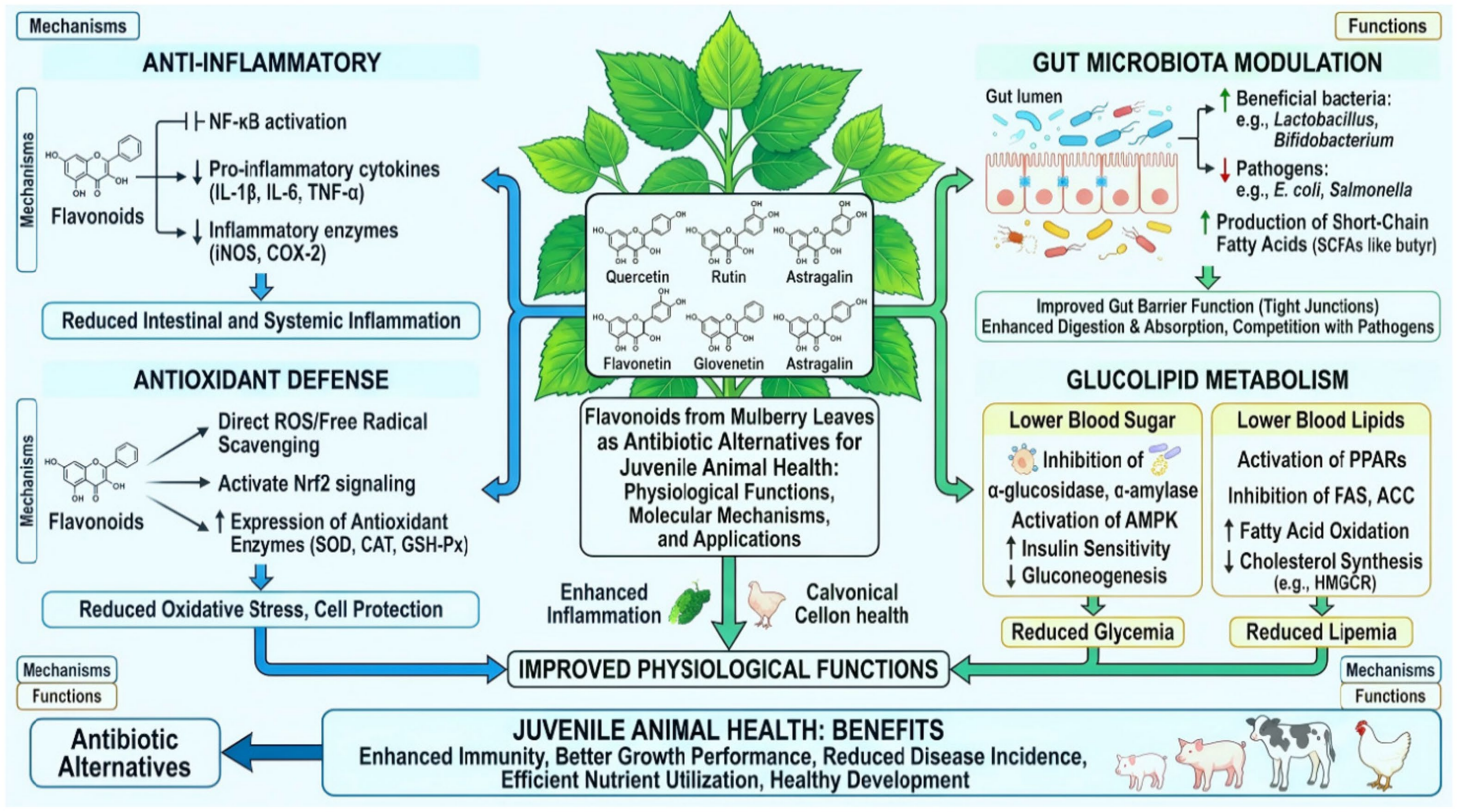
Schematic diagram of the physiological mechanisms of flavonoids from mulberry leaves in animals.

In summary, FML exhibit a range of physiological functions (Figure 1), including antioxidant and anti-inflammatory activity, modulation of intestinal microbiota, improvement of nutrient digestion and absorption, and reduction of blood lipid and glucose levels, thereby contributing to overall health and performance in animals.

## 5 Potential applications of flavonoids from mulberry leaves in juvenile animal health

Numerous studies have demonstrated that FML can effectively improve the production performance of weaned piglets, calves, chicks and juvenile fish, and exert multiple biological activities including antioxidant, anti-inflammatory, immunomodulatory and intestinal protective effects. To summarize their protective and growth-promoting functions in young animals under various physiological stresses, the applications of FML in weaned piglets, calves, juvenile fish, and chicks are organized in Table 3, focusing on their sources, dosages, key beneficial effects, and related physiological and molecular mechanisms.

**Table 3:** Effects of flavonoids from mulberry leaves on growth performance and health in juvenile animals.

Animal model	Flavonoid source and form	Dosage/application	Sample Size and experimental grouping	Key effects	Mechanisms and key index changes	References
Weaned piglets	Mulberry leaf powder	2, 4, 6% in diet	120 weaned piglets, fed for 28 days, divided into 4 treatment groups: the control group was fed a basal diet, and the experimental groups were supplemented with 2, 4, and 6% mulberry leaf powder, respectively	Supplementation reduced serum D-LA and LDH levels, elevated intestinal SOD activity, and inhibited the proliferation of hindgut potential pathogenic Allisonella	Modulates intestinal microbiota structure	(70)
	Mulberry leaf powder	3, 6, 9, 12% in diet	180 Xiangcun black pigs, fed for 50 days, divided into 5 treatment groups: the control group was fed a basal diet, and the experimental groups were supplemented with 3, 6, 9, and 12% mulberry leaf powder, respectively	Increases ADG and carcass length, improves muscle tenderness, elevates PUFA content in adipose tissue, reduces F/G ratio	Enhances nutrient deposition and feed efficiency	(71)
	*Morus alba* extract	1,000 g/t in diet	48 weaned piglets, fed for 28 days, divided into 2 treatment groups: the control group was fed a basal diet, and the experimental group was supplemented with 1,000 g/t *Morus alba* extract	Decreases plasma TNF-*α*, increases IgA and IgM in weaned piglets	Alleviates inflammatory response, enhances humoral immunity	(72)
	Quercetin	250, 500, 750 mg/kg in diet	48 weaned piglets, fed for 35 days, divided into 4 treatment groups: the control group was fed a basal diet, and the experimental groups were supplemented with 250, 500, and 750 mg/kg quercetin, respectively	Reduces diarrhea rate and diarrhea index, lowers serum NF-κB and IFN-γ, enriches Lachnospiraceae and Phascolarctobacterium	Inhibits inflammatory pathways, regulates colonic microbiota	(73)
	Quercetin	100 mg/kg in diet	48 weaned piglets, fed for 21 days, divided into 3 treatment groups: the control group was fed a basal diet, the experimental groups were supplemented with 100 mg/kg quercetin, and 100 mg/kg quercetin + 4 mg/kg deoxynivalenol, respectively	Improves villus height, upregulates occludin and claudin-1, inhibits t-RIP1/p-RIP1/t-RIP3/p-RIP3/t-MLKL/p-MLKL in DON-challenged IPEC-1	Protects intestinal barrier, relieves DON-induced injury	(74)
Calves	FML	4 g/day	48 calves, fed for 60 days, divided into 4 treatment groups: the control group was fed a basal diet, and the experimental groups were supplemented with 4 g/day FML, 1 g/day *Candida tropicalis*, and 4 g/day FML + 1 g/day *Candida tropicalis*, respectively	Increases body weight, enhances apparent digestibility of crude fat, elevates GH and IGF-1 levels	Promotes nutrient digestion and growth-related hormone secretion	(75)
	FML + *Candida tropicalis*	4 g/day FML and 1 g/day *Candida tropicalis*		Improves growth performance, feed intake and nutrient digestibility	Synergistically enhances digestive and absorptive function	
	FML	3 g/day (weaned calves)	48 calves, fed for 80 days, divided into 2 treatment groups: the control group was fed a basal diet, and the experimental group was supplemented with 3 g/day FML	Raises propionate and total VFA, stimulates rumen microbial activity, increases blood IgG, IgM, IgA	Optimizes rumen fermentation, enhances immunity, relieves weaning oxidative stress	(76)
	FML	3 g/day	15 calves, fed for 35 days, divided into 3 treatment groups: the control group was fed a basal diet, and the experimental groups were supplemented with 3 g/day FML, and 3 g/day FML + 1 g/day *Candida tropicalis*, respectively	Increases ADG and feed efficiency, enhances amylase, protease and lactase activities; Following *E. coli* challenge, abnormally elevated SOD and GSH-Px activities were restored to normal levels, alongside improved fecal scores	Boosts digestive enzyme activity, mitigates oxidative stress and diarrhea	(77)
Juvenile fish	Quercetin	200, 400, 600 mg/kg in diet (carp)	216 fish, fed for 60 days, divided into 4 treatment groups: the control group was fed a basal diet, and the experimental groups were supplemented with 200, 400, and 600 mg/kg quercetin, respectively	Improves growth, immunity and antioxidant status; upregulates GPx, lysozyme and ACH50; enhances heat stress tolerance	Strengthens antioxidant and non-specific immune functions	(78)
	Quercetin	200, 400, 800, 1,600 mg/kg (*Oreochromis niloticus*)	400 fish, fed for 49 days, divided into 5 treatment groups: the control group was fed a basal diet, and the experimental groups were supplemented with 200, 400, 800, and 1,600 mg/kg quercetin, respectively	Enhances growth performance, reduces serum and whole-body lipid levels, increases crude protein and decreases crude fat	Regulates lipid metabolism, promotes protein deposition	(79)
	Quercetin	800, 1,600 mg/kg in diet (Pb-poisoned tilapia)	400 fish, fed for 28 days, divided into 5 treatment groups: the control group was fed a basal diet, and the experimental groups were supplemented with 800 mg/kg Pb, 800 mg/kg Pb + 800 mg/kg quercetin, and 800 mg/kg Pb + 1,600 mg/kg quercetin, respectively	Alleviates growth inhibition caused by lead exposure, restores hepatopancreatic antioxidant status	Antagonizes heavy metal-induced oxidative damage	(80)
	Quercetin	200, 400, 800, 1,600 mg/kg (*Oreochromis niloticus*)	400 fish, fed for 49 days, divided into 5 treatment groups: the control group was fed a basal diet, and the experimental groups were supplemented with 200, 400, 800, and 1,600 mg/kg quercetin, respectively	Increases gastrointestinal protease, lipase and amylase activities; reduces MDA and enhances T-AOC in hepatopancreas	Improves digestive function, reduces lipid peroxidation	(81)
	Mulberry leaf extract	0, 3, 6, 9, 12, 15 g/kg in diet (*Ictalurus punctatus*)	360 fish, fed for 52 days, divided into 6 treatment groups: the control group was fed a basal diet, and the experimental groups were supplemented with 3, 6, 9, 12, and 15 g/kg mulberry leaf extract, respectively	Enhances hepatic LPS, TRS, AKP, ACP, LDH, GOT activities; decreases MDA content	Improves hepatic digestive, metabolic and immune functions	(82)
Chicks	Quercetin	0.4 g/kg quercetin，0.2 g/kg vitamin E, 0.4 g/kg quercetin + 0.2 g/kg vitamin E	400 laying hens, fed for 98 days, divided into 4 treatment groups: the control group was fed a basal diet, and the experimental groups were supplemented with 0.4 g/kg quercetin, 0.2 g/kg vitamin E, and 0.4 g/kg quercetin + 0.2 g/kg vitamin E, respectively	Enhances antioxidant capacity of offspring, improves hatching and survival rates	Transfers maternal antioxidant effects	(83)
	FML	60 mg/kg, 30 mg/kg in embryonic stage	270 breeding broilers, fed for 56 days, divided into 3 treatment groups: the control group was fed a basal diet, and the experimental groups were supplemented with 30 mg/kg and 60 mg/kg FML, respectively	Upregulates Myf5 and BMP/p-Smad1/5/9, promotes muscle fiber development; low dose better in embryo, high dose better post-hatch. Promotes skeletal muscle development, increases tibia length and ADG in broiler offspring	Regulates myogenic signaling pathways and stimulates muscle and bone growth	(84)

In summary, FML have significant potential in promoting juvenile animal health by enhancing antioxidant capacity, supporting gut development, alleviating stress, and improving growth performance. Their natural antioxidant and anti-inflammatory capacities, combined with their regulatory effects on intestinal microbiota and host metabolism, suggest that FML could act as a promising auxiliary feed additive to cut down antibiotic usage for growth promotion and disease prevention in young livestock and poultry.

## 6 Conclusion

Flavonoids from mulberry leaves are natural plant-derived compounds with dose-dependent physiological functions. Proper dietary FML supplementation enhances animal growth and production performance, showing great potential as partial antibiotic alternatives to improve animal health and immunity as well as prevent diseases in animal breeding. Nevertheless, systematic research on FML application in juvenile animals is insufficient. Existing studies mainly concentrate on weaned piglets, while relevant comprehensive trials on calves, broilers and aquatic larvae are scarce, and cross-species comparisons regarding FML absorption, optimal dosage and physiological responses are rarely reported. Future multi-species studies on young livestock and aquatic animals are required to clarify the underlying mechanisms of FML and lay a theoretical basis for its wide application as a novel feed additive in modern animal husbandry.

## References

[1] Kariuki JW Jacobs J Ngogang MP Howland O. Antibiotic use by poultry farmers in Kiambu County, Kenya: exploring practices and drivers of potential overuse. Antimicrob Resist Infect Control. (2023) 12:3. doi: 10.1186/s13756-022-01202-y, 36604700 PMC9817392

[2] Meale SJ Chaucheyras-Durand F Berends H Guan LL Steele MA. From pre- to postweaning: transformation of the young calf's gastrointestinal tract. J Dairy Sci. (2017) 100:5984–95. doi: 10.3168/jds.2016-12474, 28527800

[3] Quesnel H Resmond R Merlot E Père MC Gondret F Louveau I. Physiological traits of newborn piglets associated with colostrum intake, neonatal survival and preweaning growth. Animal. (2023) 17:100843. doi: 10.3168/jds.2016-12474 37263133

[4] Khosravi Zanjani MA Ehsani MR Ghiassi Tarzi B Sharifan A. Promoting probiotics survival by microencapsualtion with hylon starch and genipin cross-linked coatings in simulated gastro-intestinal condition and heat treatment. Iran J Pharm Res. (2018) 17:753–66. 29881432 PMC5985192

[5] Choi H Kim SW. Dietary intervention of benzoic acid for intestinal health and growth of nursery pigs. Animals (Basel). (2024) 14:2394. doi: 10.3390/ani14162394, 39199928 PMC11350768

[6] Chen X Song W Xiong P Cheng D Wei W Zhou Q . Effects of microencapsulated plant essential oils on growth performance, immunity, and intestinal health of weaned Tibetan piglets. Front Vet Sci. (2024) 11:1456181. doi: 10.3389/fvets.2024.1456181, 39229599 PMC11368909

[7] Wang Z Yin L Liu L Lan X He J Wan F . Tannic acid reduced apparent protein digestibility and induced oxidative stress and inflammatory response without altering growth performance and ruminal microbiota diversity of Xiangdong black goats. Front Vet Sci. (2022) 9:1004841. doi: 10.3389/fvets.2022.1004841, 36187804 PMC9516568

[8] Yu YF Li HY Zhang B Wang JW Shi XP Huang JZ . Nutritional and functional components of mulberry leaves from different varieties: evaluation of their potential as food materials. Int J Food Prop. (2018) 21:1495–507. doi: 10.1080/10942912.2018.1489833

[9] Liu Y Xiao Y Xie J Peng Y Li F Chen C . Dietary supplementation with flavonoids from mulberry leaves improves growth performance and meat quality, and alters lipid metabolism of skeletal muscle in a Chinese hybrid pig. Anim Feed Sci Technol. (2022) 285:115211. doi: 10.1016/j.anifeedsci.2022.115211

[10] Li MW Hassan FU Tang ZH Peng LJ Liang X Li LL . Mulberry leaf flavonoids improve milk production, antioxidant, and metabolic status of water buffaloes. Front Vet Sci. (2020) 7:599. doi: 10.3389/fvets.2020.00599, 33102551 PMC7500204

[11] Chen JJ Li XR. Hypolipidemic effect of flavonoids from mulberry leaves in triton WR-1339 induced hyperlipidemic mice. Asia Pac J Clin Nutr. (2007) 16:290–4. 17392121

[12] Ge Q Chen L Tang M Zhang S Liu LL Gao L . Analysis of mulberry leaf components in the treatment of diabetes using network pharmacology. Eur J Pharmacol. (2018) 833:50–62. doi: 10.1016/j.ejphar.2018.05.021 29782863

[13] Chan EWC Wong SK Tangah J Inoue T Chan HT. Phenolic constituents and anticancer properties of *Morus alba* (white mulberry) leaves. J Integr Med. (2020) 18:189–95. doi: 10.1016/j.joim.2020.02.006, 32115383

[14] Chen D Chen X Tu Y Wang B Lou C Ma T . Effects of mulberry leaf flavonoid and resveratrol on methane emission and nutrient digestion in sheep. Anim Nutr. (2015) 1:362–7. doi: 10.1016/j.aninu.2015.12.008, 29767046 PMC5940990

[15] Qadir R Anwar F Gilani MA Zahoor S Rehman MMU Mustaqeem M. RSM/ANN based optimized recovery of phenolics from mulberry leaves by enzyme-assisted extraction. Czech J Food Sci. (2019) 37:99–105. doi: 10.17221/147/2018-CJFS

[16] Yan CH Chen FH Yang YL Shen LW Xun XM Zhang ZA . Biochemical and protein nutritional potential of mulberry (*Morus alba* L.) leaf: partial substitution improves the nutrition of conventional protein. J Sci Food Agric. (2024) 104:2204–14. doi: 10.1002/jsfa.13103, 37934077

[17] Liu YY Peng YL Chen C Ren HB Zhu J Deng Y . Flavonoids from mulberry leaves inhibit fat production and improve fatty acid distribution in adipose tissue in finishing pigs. Anim Nutr. (2024) 16:147–57. doi: 10.1016/j.aninu.2023.11.003, 38357574 PMC10864206

[18] Ju WT Kwon OC Kim HB Sung GB Kim HW Kim YS. Qualitative and quantitative analysis of flavonoids from 12 species of Korean mulberry leaves. J Food Sci Technol. (2018) 55:1789–96. doi: 10.1007/s13197-018-3093-2, 29666531 PMC5897299

[19] Kim DS Kim SH Lee GJ Kim HK. Antioxidant activities and polyphenol content of *Morus alba* leaf extracts collected from varying regions. Planta Med. (2014) 80:1476–7. doi: 10.3892/br.2014.294 PMC4106594 25054010

[20] Kumar S Pandey AK. Chemistry and biological activities of flavonoids: an overview. Sci World J. (2013):162750. doi: 10.1155/2013/162750 PMC3891543 24470791

[21] Chiarello DI Abad C Rojas D Toledo F Vázquez CM Mate A . Oxidative stress: Normal pregnancy versus preeclampsia. Biochim Biophys Acta Mol basis Dis. (2020) 1866:165354. doi: 10.1016/j.bbadis.2018.12.005, 30590104

[22] Huang YJ Nan GX. Oxidative stress-induced angiogenesis. J Clin Neurosci. (2019) 63:13–6. doi: 10.1016/j.jocn.2019.02.019, 30837109

[23] Cao G Sofic E Prior RL. Antioxidant and prooxidant behavior of flavonoids: structure-activity relationships. Free Radic Biol Med. (1997) 22:749–60. doi: 10.1016/s0891-5849(96)00351-6, 9119242

[24] Hassanpour SH Doroudi A. Review of the antioxidant potential of flavonoids as a subgroup of polyphenols and partial substitute for synthetic antioxidants. Avicenna J Phytomed. (2023) 13:354–76. doi: 10.22038/AJP.2023.21774, 37663389 PMC10474916

[25] Liu C Huang H Chen Y Zhou Y Meng T Tan B . Dietary supplementation with mulberry leaf flavonoids and carnosic acid complex enhances the growth performance and antioxidant capacity via regulating the p38 MAPK/Nrf2 pathway. Front Nutr. (2024) 11:1428577. doi: 10.3389/fnut.2024.1428577, 39139650 PMC11319276

[26] Zhang XZ Geng AP Cao D Dugarjaviin M. Identification of mulberry leaf flavonoids and evaluating their protective effects on H2O2-induced oxidative damage in equine skeletal muscle satellite cells. Front Mol Biosci. (2024) 11:1353387. doi: 10.3389/fmolb.2024.1353387, 38650596 PMC11033687

[27] Zheng QH Feng K Zhong WT Tan WJ Rengaowa S Hu WZ. Investigating the hepatoprotective properties of mulberry leaf flavonoids against oxidative stress in HepG2 cells. Molecules. (2024) 29:2597. doi: 10.3390/molecules29112597, 38893475 PMC11173602

[28] Tuñón MJ García-Mediavilla MV Sánchez-Campos S González-Gallego J. Potential of flavonoids as anti-inflammatory agents: modulation of pro-inflammatory gene expression and signal transduction pathways. Curr Drug Metab. (2009) 10:256–71. doi: 10.2174/138920009787846369, 19442088

[29] Vauzour D Vafeiadou K Rodriguez-Mateos A Rendeiro C Spencer JPE. The neuroprotective potential of flavonoids: a multiplicity of effects. Genes Nutr. (2008) 3:115–26. doi: 10.1007/s12263-008-0091-4, 18937002 PMC2593006

[30] Chen L Teng H Jia Z Battino M Miron A Yu ZL . Intracellular signaling pathways of inflammation modulated by dietary flavonoids: the most recent evidence. Crit Rev Food Sci Nutr. (2018) 58:2908–24. doi: 10.1080/10408398.2017.1345853, 28682647

[31] Al-Khayri JM Sahana GR Nagella P Joseph BV Alessa FM Al-Mssallem MQ. Flavonoids as potential anti-inflammatory molecules: a review. Molecules. (2022) 27:2901. doi: 10.3390/molecules27092901, 35566252 PMC9100260

[32] Martinez D Palmer C Simar D Cameron BA Nguyen N Aggarwal V . Characterisation of the cytokine milieu associated with the up-regulation of IL-6 and suppressor of cytokine 3 in chronic hepatitis C treatment non-responders. Liver Int. (2015) 35:463–72. doi: 10.1111/liv.12473, 24461080

[33] Duan YH Dai HY An YC Cheng L Shi L Lv YL . Mulberry leaf flavonoids inhibit liver inflammation in type 2 diabetes rats by regulating TLR4/MyD88/NF-κB signaling pathway. Evid Based Complement Alternat Med. (2022) 2022:3354062. doi: 10.1155/2022/3354062, 35845591 PMC9279020

[34] Shi Y Zhong L Fan Y Zhang J Zhong H Liu X . The protective effect of mulberry leaf flavonoids on high-carbohydrate-induced liver oxidative stress, inflammatory response and intestinal microbiota disturbance in *monopterus albus*. Antioxidants. (2022) 11:976. doi: 10.3390/antiox11050976, 35624840 PMC9137898

[35] Sun X Zhou X He WM Sun W Xu Z. Co-housing and fecal microbiota transplantation: technical support for tcm herbal treatment of extra-intestinal diseases based on gut microbial ecosystem remodeling. Drug Des Devel Ther. (2023) 17:3803–31. doi: 10.2147/DDDT.S443462, 38155743 PMC10753978

[36] Oskoueian E Abdullah N Oskoueian A. Effects of flavonoids on rumen fermentation activity, methane production, and microbial population. Biomed Res Int. (2013) 2013:349129. doi: 10.1155/2013/349129, 24175289 PMC3794516

[37] Zou LJ Shao YR Xiong X Ying YL. Current advances on the regulation of intestinal mucosal barrier function in weaned piglets by flavonoids (in Chinese). Sci Sinica. (2025) 55:30–44. doi: 10.1360/SS-2024-0238

[38] Li M Hassan F Peng L Xie H Liang X Huang J . Mulberry flavonoids modulate rumen bacteria to alter fermentation kinetics in water buffalo. Peerj. (2022) 10:e14309. doi: 10.7717/peerj.14309, 36536626 PMC9758972

[39] Bi YL Yang CT Diao QY Tu Y. Effects of dietary supplementation with two alternatives to antibiotics on intestinal microbiota of preweaned calves challenged with *Escherichia coli* K99. Sci Rep. (2017) 7:5439. doi: 10.1038/s41598-017-05376-z, 28710379 PMC5511211

[40] Zhu CX Liu GX Gu XK Zhang TQ Xia AJ Zheng Y . Effects of quercetin on the intestinal microflora of freshwater dark sleeper. *Odontobutis potamophila*. Antioxidants (Basel). (2022) 11:2015. doi: 10.3390/antiox11102015, 36290739 PMC9598073

[41] Zhong YZ Song B Zheng CB Zhang SY Yan ZM Tang ZY . Flavonoids from mulberry leaves alleviate lipid dysmetabolism in high fat diet-fed mice: involvement of gut microbiota. Microorganisms. (2020) 8:860. doi: 10.3390/microorganisms8060860, 32517288 PMC7355566

[42] Ma T Chen DD Tu Y Zhang NF Si BW Diao QY. Dietary supplementation with mulberry leaf flavonoids inhibits methanogenesis in sheep. Anim Sci J. (2017) 88:72–8. doi: 10.1111/asj.12556, 27112278

[43] Zhang LW Su SL Zhu Y Guo JM Guo S Qian DW . Mulberry leaf active components alleviate type 2 diabetes and its liver and kidney injury in db/db mice through insulin receptor and TGF-β/Smads signaling pathway. Biomed Pharmacother. (2019) 112:108675. doi: 10.1016/j.biopha.2019.108675, 30780108

[44] Cai SY Sun W Fan YX Guo X Xu GY Xu TH . Effect of mulberry leaf (*Folium Mori*) on insulin resistance via IRS-1/PI3K/Glut-4 signalling pathway in type 2 diabetes mellitus rats. Pharm Biol. (2016) 54:2685–91. doi: 10.1080/13880209.2016.1178779, 27158744

[45] Gouveia H Urquiza-Martínez MV Manhaes-de-Castro R Costa-de-Santana BJR Villarreal JP Mercado-Camargo R . Effects of the treatment with flavonoids on metabolic syndrome components in humans: a systematic review focusing on mechanisms of action. Int J Mol Sci. (2022) 23:8344. doi: 10.3390/ijms23158344, 35955475 PMC9369232

[46] Huang ZW Lv ZP Dai HJ Li SM Jiang JL Ye NW . Dietary mulberry-leaf flavonoids supplementation improves liver lipid metabolism and ovarian function of aged breeder hens. J Anim Physiol Anim Nutr. (2022) 106:1321–32. doi: 10.1111/jpn.13658, 34741341

[47] Feng GY Liu YY Huang RL Zhou L. Effects of mulberry leaf flavonoids on chemical composition of liver and kidney in Bama Xiang pigs (in Chinese). Chin J Anim Nutr. (2021) 33:4943–57. doi: 10.3969/j.issn.1006-267x.2021.09.012

[48] Cui H Lu TH Wang MX Zou XT Zhang Y Yang XD . Flavonoids from *Morus alba* L. leaves: optimization of extraction by response surface methodology and comprehensive evaluation of their antioxidant, antimicrobial, and inhibition of α-amylase activities through analytical hierarchy process. Molecules. (2019) 24:2398. doi: 10.3390/molecules24132398, 31261837 PMC6651629

[49] Kobus-Cisowska J Szczepaniak O Szymanowska-Powalowska D Piechocka J Szulc P Dziedzinski M. Antioxidant potential of various solvent extract from *Morus alba* fruits and its major polyphenols composition. Cienc Rural. (2020) 50:e20190371. doi: 10.1590/0103-8478cr20190371

[50] Vilkhu K Mawson R Simons L Bates D. Applications and opportunities for ultrasound assisted extraction in the food industry - a review. Innov Food Sci Emerg Technol. (2008) 9:161–9. doi: 10.1016/j.ifset.2007.04.014

[51] Ouédraogo JCW Dicko C Kini FB Bonzi-Coulibaly YL Dey ES. Enhanced extraction of flavonoids from *Odontonema strictum* leaves with antioxidant activity using supercritical carbon dioxide fluid combined with ethanol. J Supercrit Fluids. (2018) 131:66–71. doi: 10.1016/j.supflu.2017.08.017

[52] Chan CH Yusoff R Ngoh GC Kung FWL. Microwave-assisted extractions of active ingredients from plants. J Chromatogr A. (2011) 1218:6213–25. doi: 10.1016/j.chroma.2011.07.040, 21820119

[53] Guo XL Ren TZ Li HB Lu QX Di X. Antioxidant activity and mechanism exploration for microwave-assisted extraction of flavonoids from *Scutellariae Radix* using natural deep eutectic solvent. Microchem J. (2024) 200:110300. doi: 10.1016/j.microc.2024.110300

[54] Wang J Wu FA Zhao H Liu L Wu QS. Isolation of flavonoids from mulberry (*Morus alba* L.) leaves with macroporous resins. Afr J Biotechnol. (2008) 7:2147–55.

[55] Jia DD Li SF Gu ZP. Preparative isolation of flavonoids from mulberry (*Morus alba* L.) leaves by macroporous resin adsorption. J Food Process Eng. (2011) 34:1319–37. doi: 10.1111/j.1745-4530.2009.00427.x

[56] Echeverría J Opazo J Mendoza L Urzúa A Wilkens M. Structure-activity and lipophilicity relationships of selected antibacterial natural flavones and flavanones of chilean flora. Molecules. (2017) 22:608. doi: 10.3390/molecules22040608, 28394271 PMC6154607

[57] Suh MG Choi HS Cho K Park SS Kim WJ Suh HJ . Anti-inflammatory action of herbal medicine comprised of Scutellaria baicalensis and *Chrysanthemum morifolium*. Biosci Biotechnol Biochem. (2020) 84:1799–809. doi: 10.1080/09168451.2020.1769464, 32448093

[58] Xiao ZC He LL Hou XH Wei JP Ma XY Gao ZH . Relationships between structure and antioxidant capacity and activity of glycosylated flavonols. Foods. (2021) 10:849. doi: 10.3390/foods10040849, 33919682 PMC8070355

[59] Alsharairi NA. Quercetin derivatives as potential therapeutic agents: An updated perspective on the treatment of nicotine-induced non-small cell lung cancer. Int J Mol Sci. (2023) 24:15208. doi: 10.3390/ijms242015208, 37894889 PMC10607898

[60] Tabeshpour J Hosseinzadeh H Hashemzaei M Karimi G. A review of the hepatoprotective effects of hesperidin, a flavanon glycoside in citrus fruits, against natural and chemical toxicities. Daru. (2020) 28:305–17. doi: 10.1007/s40199-020-00344-x, 32277430 PMC7214587

[61] Olicheva V Beloborodov V Sharifi S Dubrovskaya A Zhevlakova A Selivanova I . Dihydroquercetin and related flavonoids in antioxidant formulations with α-tocopherol. Int J Mol Sci. (2025) 26:5659. doi: 10.3390/ijms26125659, 40565124 PMC12193696

[62] Zhang Y Yu J Dong XD Ji HY. Research on characteristics, antioxidant and antitumor activities of dihydroquercetin and its complexes. Molecules. (2018) 23:20. doi: 10.3390/molecules23010020, 29271900 PMC5943956

[63] Okkay U Okkay IF Cicek B Aydin IC Ozkaraca M. Hepatoprotective and neuroprotective effect of taxifolin on hepatic encephalopathy in rats. Metab Brain Dis. (2022) 37:1541–56. doi: 10.1007/s11011-022-00952-3, 35298730

[64] Irianti MI Vincken JP van Dinteren S ter Beest E Pos KM Araya-Cloutier C. Prenylated isoflavonoids from *Fabaceae* against the NorA efflux pump in *Staphylococcus aureus*. Sci Rep. (2023) 13:22548. doi: 10.1038/s41598-023-48992-8, 38110428 PMC10728173

[65] Lee WK Kim SJ. Protective effects of isoflavones on alcoholic liver diseases: computational approaches to investigate the inhibition of ALDH2 with isoflavone analogues. Front Mol Biosci. (2023) 10:1147301. doi: 10.3389/fmolb.2023.1147301, 36923641 PMC10009234

[66] Leonetti D Soleti R Clere N Vergori L Jacques C Duluc L . Extract enriched in flavan-3-ols and mainly procyanidin dimers improves metabolic alterations in a mouse model of obesity-related disorders partially via estrogen receptor alpha. Front Pharmacol. (2018) 9:406. doi: 10.3389/fphar.2018.00406, 29740325 PMC5928481

[67] Nawrot-Hadzik I Matkowski A Hadzik J Dobrowolska-Czopor B Olchowy C Dominiak M . Proanthocyanidins and flavan-3-ols in the prevention and treatment of periodontitis-antibacterial effects. Nutrients. (2021) 13:165. doi: 10.3390/nu13010165, 33430257 PMC7825738

[68] Mirossay L Varinská L Mojzis J. Antiangiogenic effect of flavonoids and chalcones: an update. Int J Mol Sci. (2018) 19:27. doi: 10.3390/ijms19010027 PMC5795978 29271940

[69] Ouyang Y Li JJ Chen XY Fu XY Sun S Wu Q. Chalcone derivatives: role in anticancer therapy. Biomolecules. (2021) 11:894. doi: 10.3390/biom11060894, 34208562 PMC8234180

[70] Ma J Wang J Jin X Liu S Tang S Zhang Z . Effect of dietary supplemented with mulberry leaf powder on growth performance, serum metabolites, antioxidant property and intestinal health of weaned piglets. Antioxidants (Basel). (2023) 12:307. doi: 10.3390/antiox12020307, 36829865 PMC9952558

[71] Liu YY Li YH Peng YL He JH Xiao DF Chen C . Dietary mulberry leaf powder affects growth performance, carcass traits and meat quality in finishing pigs. J Anim Physiol Anim Nutr. (2019) 103:1934–45. doi: 10.1111/jpn.13203, 31478262

[72] Song M Wang CP Deng D Liu ZC Chen QY Cui YY . Effects of mulberry leaf extract on growth performance, apparent nutrient digestibility, antioxidant capacity and immune function of weaned piglets (in Chinese). Chin J Anim Nutr. (2022) 34:3537–46. doi: 10.3969/j.issn.1006-267x.2022.06.016

[73] Mao Y Yang Q Liu J Fu Y Zhou S Liu J . Quercetin increases growth performance and decreases incidence of diarrhea and mechanism of action in weaned piglets. Oxidative Med Cell Longev. (2024) 2024:5632260. doi: 10.1155/2024/5632260, 39139212 PMC11321896

[74] Liu JH Zhou MH Xu QL Lv QQ Guo JJ Qin X . Quercetin ameliorates deoxynivalenol-induced intestinal injury and barrier dysfunction associated with inhibiting necroptosis signaling pathway in weaned pigs. Int J Mol Sci. (2023) 24:15172. doi: 10.3390/ijms242015172, 37894853 PMC10607508

[75] Zhang LY Qu PB Tu Y Yang CT Diao QY. Effects of flavonoids from mulberry leaves and *Candida tropicalis* on performance and nutrient digestibility in calves. Kafkas Univ Vet Fak Derg. (2017) 23:473–9. doi: 10.9775/kvfd.2016.17111

[76] Kong LX Yang CT Dong LF Diao QY Si BW Ma JN . Rumen fermentation characteristics in pre- and post-weaning calves upon feeding with mulberry leaf flavonoids and *Candida tropicalis* individually or in combination as a supplement. Animals. (2019) 9:990. doi: 10.3390/ani9110990, 31752155 PMC6912756

[77] Wang B Yang CT Diao QY Tu Y. The influence of mulberry leaf flavonoids and *Candida tropicalis* on antioxidant function and gastrointestinal development of preweaning calves challenged with *Escherichia coli* O141:K99. J Dairy Sci. (2018) 101:6098–108. doi: 10.3168/jds.2017-13957, 29680656

[78] Armobin K Ahmadifar E Adineh H Samani MN Kalhor N Yilmaz S . Quercetin application for common carp (*Cyprinus carpio*): I. Effects on growth performance, humoral immunity, antioxidant status, immune-related genes, and resistance against heat stress. Aquac Nutr. (2023) 2023:1–10. doi: 10.1155/2023/1168262, 36860974 PMC9973228

[79] Zhai SW Liu SL. Effects of dietary quercetin on growth performance, serum lipids level and body composition of tilapia. Ital J Anim Sci. (2013) 12:1168262. doi: 10.4081/ijas.2013.e85

[80] Zhai SW Liu SL Chen XH. Effects of quercetin on alleviating dietary lead (Pb)-induced growth retardation and oxidative stress in juvenile tilapia (*Oreochromis niloticus*). Isr J Aquac Bamidgeh. (2015) 67:1080. doi: 10.46989/001c.20769

[81] Zhai SW Liu SL. Effects of dietary quercetin on the growth performance, digestive enzymes and antioxidant potential in the hepatopancreas of tilapia (*Oreochromis niloticus*). Isr J Aquac Bamidgeh. (2014) 66:1038. doi: 10.46989/001c.20770

[82] Zhou SS Lin H Kong LM Ma JR Long ZY Qin HH . Effects of mulberry leaf extract on the liver function of juvenile spotted sea bass (*Lateolabrax maculatus*). Aquac Nutr. (2023) 2023:2892463. doi: 10.1155/2023/2892463, 37908498 PMC10615578

[83] Amevor FK Cui ZF Du XX Ning ZF Deng X Xu D . Synergy between dietary quercetin and vitamin E supplementation in aged hen's diet improves hatching traits, embryo quality, and antioxidant capacity of chicks hatched from eggs subjected to prolonged storage. Front Physiol. (2022) 13:873551. doi: 10.3389/fphys.2022.873551, 35480036 PMC9035936

[84] Huang ZW Dai HJ Li SM Wang Z Wei QW Ning ZH . Maternal supplementation with mulberry-leaf flavonoids improves the development of skeletal muscle in the offspring of chickens. Anim Nutr. (2024) 18:72–83. doi: 10.1016/j.aninu.2024.04.005, 39035983 PMC11260304

